# ASSOCIATIONS BETWEEN ATTITUDES TOWARDS AGING AND CLINICAL DECISIONS AMONGST INTERNAL MEDICINE RESIDENTS

**DOI:** 10.1093/geroni/igac059.2765

**Published:** 2022-12-20

**Authors:** Brent Schell, Megan Carr, Jesse Katz, Maryam Hasan

**Affiliations:** Baystate Medical Center, Springfield, Massachusetts, United States; High Point University, High Point, North Carolina, United States; Baystate Medical Center, Springfield, Massachusetts, United States; Baystate Medical Center, Springfield, Massachusetts, United States

## Abstract

It has been shown that physicians-in-training can develop negative attitudes towards caring for older patients. Little is known about how these attitudes develop throughout training or if they are associated with ageist clinical decisions. In this pilot study, Internal Medicine (IM) residents completed the Expectations Regarding Aging (ERA-12) survey, a validated instrument that assesses expectations regarding physical health, mental health, and cognitive function in older adults. Residents then completed a survey that included simulated clinical scenarios as part of an educational intervention on Ageism. Of the 72 residents invited to participate, 48 residents (67%) completed the ERA-12 survey. Responses were obtained from all training levels including 24 PGY-1 (50%), 10 PGY-2 (21%), and 14 PGY-3 (29%). When grouped by training year, there were no statistically significant differences found with regards to total ERA-12 scores or expectations regarding physical health, cognitive function, or mental health of older adults. When grouped by responses to clinical scenarios, residents with more biased clinical responses were associated with lower total ERA scores (73 vs. 91, p = 0.015). Those with lower total ERA scores indicated they were inclined to initiate CODE STATUS discussions earlier with older adults and were less inclined to consider coexisting depression. Despite limitations in sample size and the subjective nature of simulated clinical scenarios, this pilot study suggests a possible association between expectations regarding aging and clinical decision-making. More research is needed to assess the strength of this association and if it can be modified through educational initiatives.

